# The Effect of Exercise Intervention on Disability and Kinesiophobia in a Retired Athlete With Old Patella Fracture: A Case Report

**DOI:** 10.3389/fpsyg.2021.744433

**Published:** 2021-11-12

**Authors:** Yuqin Su, Li Huang, Haowei Liu, Shifan Chen, Li Peng

**Affiliations:** ^1^College of Physical Education, Chongqing University of Posts and Telecommunications, Chongqing, China; ^2^College of Physical Education, Southwest University, Chongqing, China; ^3^Key Lab of Physical Fitness Evaluation and Motor Function Monitoring, Southwest University, Chongqing, China; ^4^Electrical Engineering and Automation Department, College of Automation, Chongqing University of Posts and Telecommunications, Chongqing, China

**Keywords:** retired athlete, exercise intervention, kinesiophobia, disability, patella fracture

## Abstract

**Objectives:** To evaluate the effect of exercise intervention on disability, pain, and kinesiophobia in a retired athlete with old patella fracture.

**Methods:** A 34-year-old retired football player with old patella fracture conducted the exercise intervention for 12 weeks, 1 h each time, three times a week. the retired football player completed the Lysholm Knee Score (LKS), Visual Analog Scale (VAS), and the Tampa Scale for Kinesiophobia (TSK) were measured at pre-intervention, mid-intervention, and post-intervention.

**Results:** Based on the functional training perspective, the retired athlete was subjected to two stages of exercise intervention for a total of 12 weeks. The patient's LKS score increased from 76 to 95, and the pain level of various physical states was relieved. When walking, the VAS score was reduced from 3 to 1, and when running, the VAS score was reduced from 5 to 2. Jumping VAS score for actions was reduced from 6 to 3, and the VAS score for of daily life activities was reduced from 3 points to 2. The patient's TSK score from 50 to 37.

**Conclusion:** A 12-week exercise intervention could improve knee joint function, relieve pain and relieve kinesiophobia.

## Introduction

The patella is an important part of the knee joint. It creates good mechanical conditions for knee extension and plays an important role in lower limb activities and walking. Patella fractures comprise 1% of all fractures (Henrichsen et al., 2018). It mostly caused by direct or indirect violence. Patella fractures negatively impacts the patient's health and quality of life, and brings long-term economic burden. Long-term pain and dysfunction often resulting in a downward spiral of negative physical, social and psychological consequences, and among the many biopsychosocial factors which contribute to the experience and impact of pain, negative or maladaptive psychological factors (e.g., fear) are among the most important (Luque-Suarez et al., 2019). This kinesiophobia can precipitate changes to cortical networks that perceive and regulate motor functions, and receiving evidence-based treatments that were initially effective many patients report recurrent or persistent symptoms for years after the initial diagnosis (Borisovskaya, 2020). For athletes, the impact of sports fear will be more obvious. Fear of reinjury after a sports injury can negatively affect the recovery of physical impairments, reduce self-report function, and prevent a successful return to sport (Hsu et al., 2017). Untreated psychological factors could be a possible explanation for persistent symptoms and poor treatment outcome. Psychological factors affect the rehabilitation of patients with chronic musculoskeletal pain. Studies have pointed out that kinesiophobia and catastrophizing have proved to rule an important role in the chronicity of other musculoskeletal disorders (Domenech et al., 2013).

Kinesiophobia is defined as an excessive, irrational, and debilitating fear of physical movement and activity resulting from a feeling of vulnerability to painful injury or reinjury (Kori et al., 1990). At present, the Tampa Scale for Kinesiophobia (TSK) is usually used to assess the degree of fear of movement in patients with chronic pain. When the TSK score is >37 points, it is judged to have Kinesiophobia (Heuts et al., 2004). Fear of movement can negatively affect the recovery of physical impairments, reduce self-report function, and prevent a successful return to sport (Hsu et al., 2017). The fear of exercise associated with old patella fractures will not only reduce the patient's physical activity level, but also have a certain impact on their physical functions. For these patients, the main purpose is to solve the fear of movement and dysfunction. And more and more evidence supporting the relationship between pain-related fear and functional disability in chronic musculoskeletal pain conditions, and the study findings underscore the importance of pain-related fear in daily functioning of patients with chronic knee pain (Heuts et al., 2004). Fear of movement is also associated with pain, disability, and quality of life. Many studies have also studied the relationship between fear of movement and the severity of pain, and found that there is a clear correlation between fear of exercise and the generation and severity of pain. The higher the pain score, the higher the degree of fear (Karayannis et al., 2013; Fagevik et al., 2016). At the same time, fear of movement is also associated with pain, disability, and reduced quality of life (Oskay et al., 2017; Luque-Suarez et al., 2019).

At present, cognitive behavioral therapy (Archer et al., 2016), exercise intervention (Hanel et al., 2020), multidisciplinary intervention (Monticone et al., 2013), multidisciplinary cognitive behavioral rehabilitation (Monticone et al., 2016) have been used to improve patients' kinesiophobia and prompt patients to resume functional exercise and daily activities. Exercise intervention is the preferred way for the management of chronic knee pain to improve dysfunction, relieve pain, and reduce the degree of fear of exercise in patients. Exercise intervention has well-documented anti-inflammatory effects, and preliminary studies show that exercise can reduce systemic inflammation, which in turn may reduce chronic pain (Sluka et al., 2018). Simultaneously, exercise intervention could promote cognitive restructuring by decreasing fear-avoidance and pain catastrophizing and improving self-efficacy (Nijs et al., 2015; Booth et al., 2017). A study has found that a 6-week team-based rehabilitation program for Total knee arthroplasty patients, the rehabilitation program included aerobic exercise and lower-extremity muscle strengthening exercises, and the result showed that the applied group-based intervention led to a significant improvement in functional ambulation and physical activity (Krumov et al., 2019). Another study conducted 12 weeks of Pilates exercise intervention on 64 patients with non-specific low back pain, and the results showed that pilates intervention in patients with chronic non-specific low back pain is effective in the management of disability, pain, and kinesiophobia (Cruz-Díaz et al., 2018). Also, a study conducted 3 months of exercise intervention for patients with patellofemoral pain, exercise intervention can improve knee joint pain, panic disorder, and knee self-efficacy (Hott et al., 2019). At present, only very little literature reported the effects of an exercise intervention on the physical function, disability and pain, and kinesiophobia of patients with chronic knee pain. At the same time, there is still a lack of exercise intervention for kinesiophobia, physical function, and pain in patients with old patella fractures. Exercise intervention may be an effective treatment to reduce fear of movement, but more research is needed to expand the effect of exercise intervention. In addition, whether exercise intervention can truly improve panic disorder, physical function, and pain in patients with chronic knee pain is a topic that remains to be verified. Still, no research is to conduct individual targeted assessment and training of individuals, especially for retired athletes with chronic knee pain. Then the purpose of this case report is to explore the effects of an exercise intervention on the function, disability, pain and kinesiophobia of retired athletes with chronic knee pain.

## Materials and Methods

### Case Description

**Figure 1:**
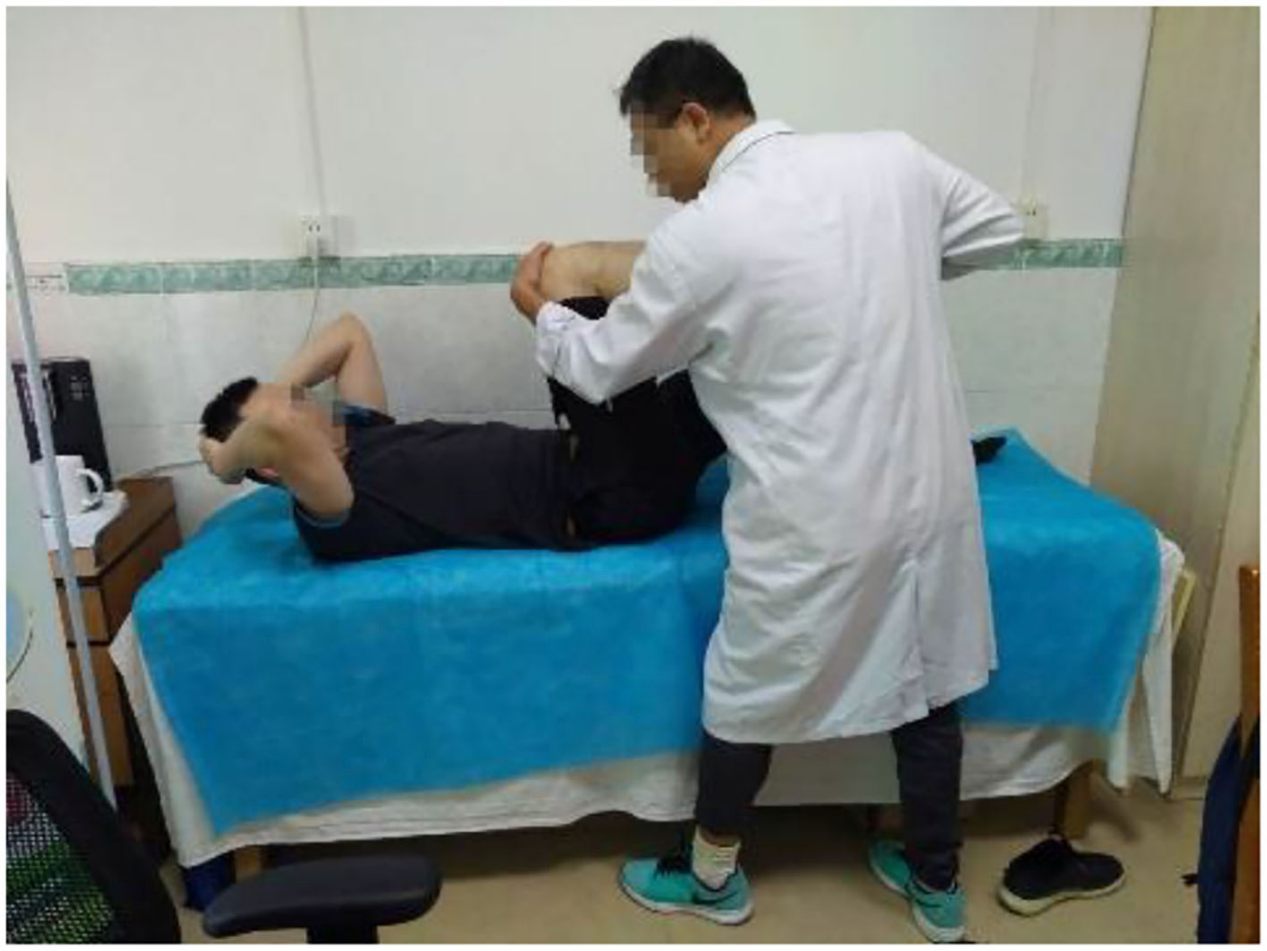
The patient's physical examination was performed.

A 34-year-old retired football player started to practice football in 2001 and now worked in a gym as a personal trainer. In March 2004, the patient immediately felt pain in his right knee, and his right leg was unable to take off after a quick long jump without warming up enough. There was no swelling of the knee joint at the time of the injury. The patient felt his legs were soft, unable to exert force, and felt deep in the restricted position but he could continue to train. In December 2004, an MRI examination was diagnosed with an osteochondral contusion of the lateral femoral condyle. Sodium hyaluronate was injected in 2006. On the advice of doctors, he retired in April 2006. From 2006 to 2013, the patient mainly sold sports equipment and worked as a part-time model. During the past few years, the patient did not treat with relevant functional rehabilitation and was limited to physical activities of daily life. He has been a fitness instructor since 2013, the patient has been continuously undergoing acupuncture, physiotherapy and small needle knife treatment, but the knee joint pain still exists after the treatment, and at the same time, the knee joint pain worsens after each training session. The patient complained that there was no obvious pain in the right knee in daily life, but the knee joint would be uncomfortable when it was cold. The right knee has obvious downhill pain, deep pain, blurred pain, unable to take off normally and participating in strenuous exercises of the lower limbs, fearing to be injured again, dare not do high-intensity and difficult movements in daily life and training. On April 24, 2019, a Physical and Rehabilitation Medicine (PRM) physician performed physical examinations (Figure 1), and a PRM physician experienced in musculoskeletal ultrasound diagnostic used Samsung HM70A model 5 18 Hz frequency probe to examine the patient's painful knee joint. The results showed that active range of motion (AROM) of the right hip joint: flexion-extension-abduction-adduction-external rotation-internal rotation were 110°-12°-33°-18°-40°-35°; left side hip joint AROM flexion-extension-abduction-adduction-external rotation-internal rotation were 115°-9°-25°-20°-36°-35°. Both sides hip joint extension and abduction were insufficient; Right and left knee flexion were 115° and 121°; right and left ankle dorsiflexion-plantar flexion were 15°-35° and 9°-30°, which indicated the left ankle AROM of the dorsiflexion joint was insufficient. The flexion and extension of the knee joints of both lower extremities were tested on grade 5 with normal muscle strength. The patient had no other medical or family history.

**Figure 2:**
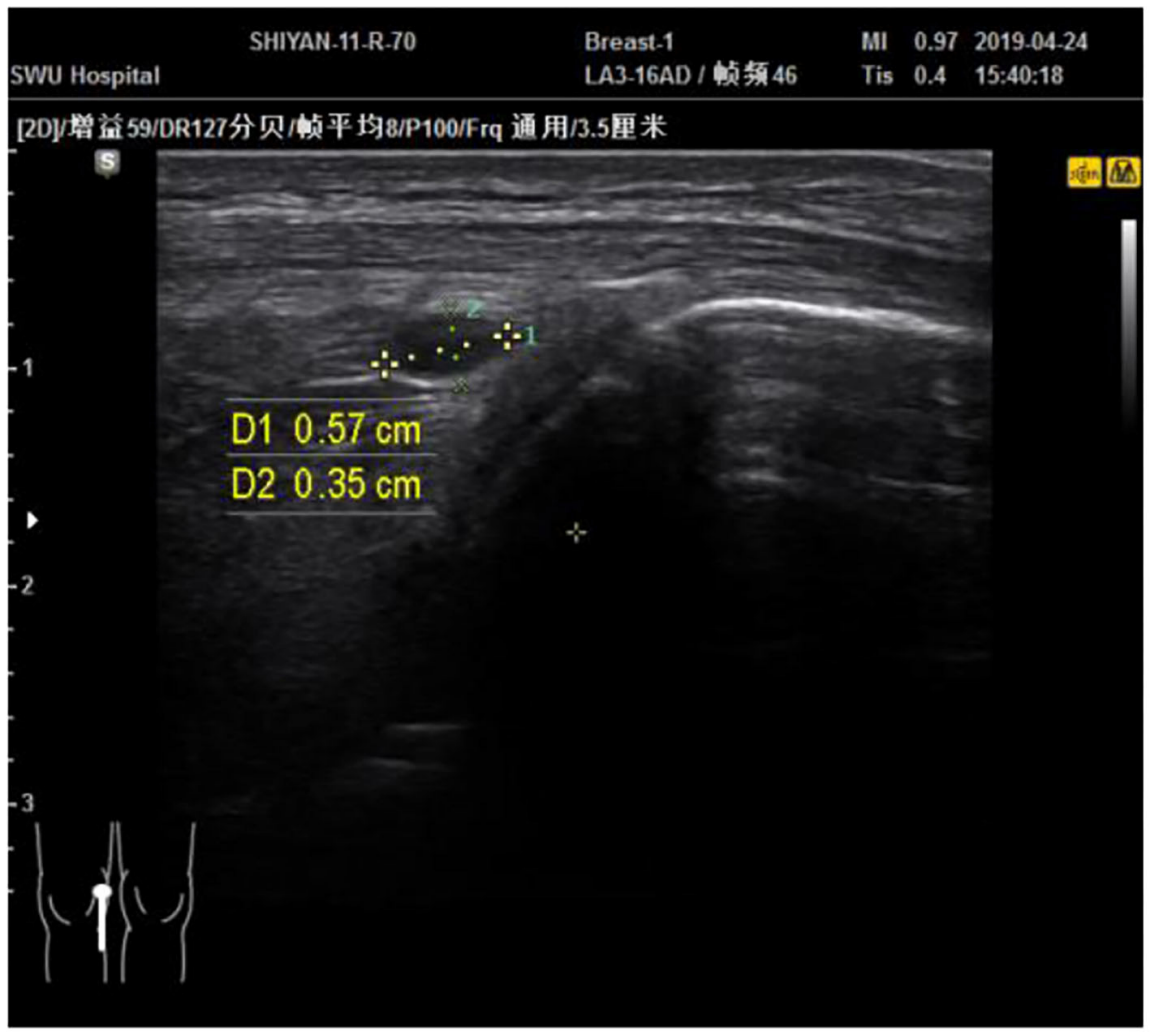
The patient's deep subpatella sac effusion.

The musculoskeletal ultrasound examination showed that the continuity of the patellar cortex was interrupted. An echoless area (about 0.57^*^0.35) was visible on the deep surface of the patellar tendon (Figure 2). The inner cartilage was slightly thinner than the outer side. The diagnosis was fluid accumulation in the deep subpatellar sac and old patella fracture. The patient signed informed consent and corrective training instructions to allow the use of his personal information for this case report and promise to follow the direction of exercise intervention. The patient himself was unwilling to undergo surgical treatment and only accepted conservative treatment. The patient was approved by the ethics committee of Physical Education of Southwest University School (approval no. SWU-20180304-C1).

### Methods

The assessment of the patient with chronic knee pain was performed 1 week before exercise intervention. Medical examination, the Visual Analog Scale (VAS), Lysholm Knee Score (LKS) and Tampa Scale for Kinesiophobia (TSK). Based on the initial evaluation results, the patient underwent a multimodal intervention for a total of 12 weeks 3 times a week for 1 h each time. During the intervention, the patient was not asked to change his way of life and behavior but maintained his normal state of life, including daily activity, and continued working in the gym and giving clients 3 training sessions a week.

### Lysholm Knee Score

LKS is a popular patient-reported instrument that measures outcomes in patients with knee problems. It included 8 domains that assess instability (0–25 points), pain (0–25 points), locking (0–15 points), stair-climbing (0–10 points), swelling (0–10 points), and support (0–5 points), squatting (0–5 points), and limping (0–5 points). The score ranges from 0 to 100 points is calculated, with excellent (95–100 points), good (84–94 points), fair (65–83 points), and poor (≤64 points) (Panagopoulos et al., 2020).

### Visual Analog Scale

The visual analog scale (VAS) was used to evaluate the pain degree. It is a 10cm line segment; 0 means no pain, 10 means severe pain; the larger the number, the more severe the pain (Heller et al., 2016).

### Tampa Scale for Kinesiophobia

TSK was used to evaluate the degree of fear of movement in patients. The TSK was developed by Miller et al. (1991). It is one of the most widely-used assessment tools for kinesiophobia and has good reliability and validity (Huang et al., 2019). The TSK includes 17 items, among which 4 items (i.e., items 4, 8, 12, 16) are reverse-scored. The test's total score is the sum of the points marked by the patient, ranging from 17 to 68. The higher the score, the higher the patient's fear of movement/(re)injury. If the score is great than 37, the patient is considered to suffer from kinesiophobia (Hsu et al., 2017).

### Exercise Protocol

Pre-exercise tests found that the patient's knee joints were unstable; the function of climbing stairs was weakened; ankle range of motion was lack; knee pain was aggravated when jumping; balance ability was weakened, and kinesiophobia was indicated. The intervention was composed of two exercise protocol stages. Refer to the correction training guide for dysfunctionto improve breathing and achieve the best joint coaxiality (Inc, 2012). The principle of respiration and joint coaxiality is incorporated into the basic movement mode. According to the requirements of increasing movement and effective transfer of functions, a sports rehabilitation training program has been formulated. The exercise movements was linked with the examination findings and described in detail by the functional movement websites (https://www.functionalmovement.com) and Pilates books. The training movement is mainly on the mat or fixed equipment, and the movement form is dynamic movement and low load.

The first stage was performed in the laboratory, 60min/time, 3 times/week, a total of 8 weeks, with rehabilitation exercises conducted professionally by qualified coaches one-on-one. The exercises were mainly based on Mike Boyle's adjacent joint hypothesis (joint-by-joint approach) to strengthen the patient's ankle and hip joint function, and improve the stability of the knee joint (Cook, 2015). The main idea is to release the lower limb muscles with foam roller, especially the right quadriceps, iliotibial band, pes anserinus tendon and gluteus medius and gluteus minimus, and then strengthen the flexibility of the ankle and hip joints. Training, through kneeling ankle dorsiflexion exercises, heel raise exercises, ankle loops and bands to assist straight leg raising exercises and then strengthen the quadriceps, hamstrings, gluteus medius and gluteus minimus, and finally build movement patterns of hip flexion, single-leg squat, double-leg jump and single-leg jump. All movements were performed according to the patient's physical condition to raise and lower the order, each movement is 8–12 times/set, a total of 3 sets.

The second stage was for the patient to exercise on his own, 60 min/time, 2 times/week, for a total of 8 weeks. Based on his feelings and experience in his coaching career, the patient made a training plan for himself on the basis of the first stage. The first step was to strengthen the flexibility of the hip joint, focusing on stretching the iliopsoas muscles and quadriceps. The patient was self-assessed that the right quadriceps muscles have greater tension than the left, and he paid more attention to foam roller rolling and stretching. The second step was to improve the lower limb strength including gluteus medius, gluteus minimus, hamstrings and quadriceps. Four main movements were performed (i.e., shoulder bridge, clam opening and closing, sitting leg flexion and extension, and prone knee flexion). In addition, the patient self-assessed the right foot varus, which also increased the balance training of the ankle joint. Finally, to enhance the core strength, three main actions were performed (i.e., abdominal crunch, Russian rotation, and supine leg lift). Each movement is 12 times/set, 3 sets in total.

## Results

### LKS Results

**Figure 3:**
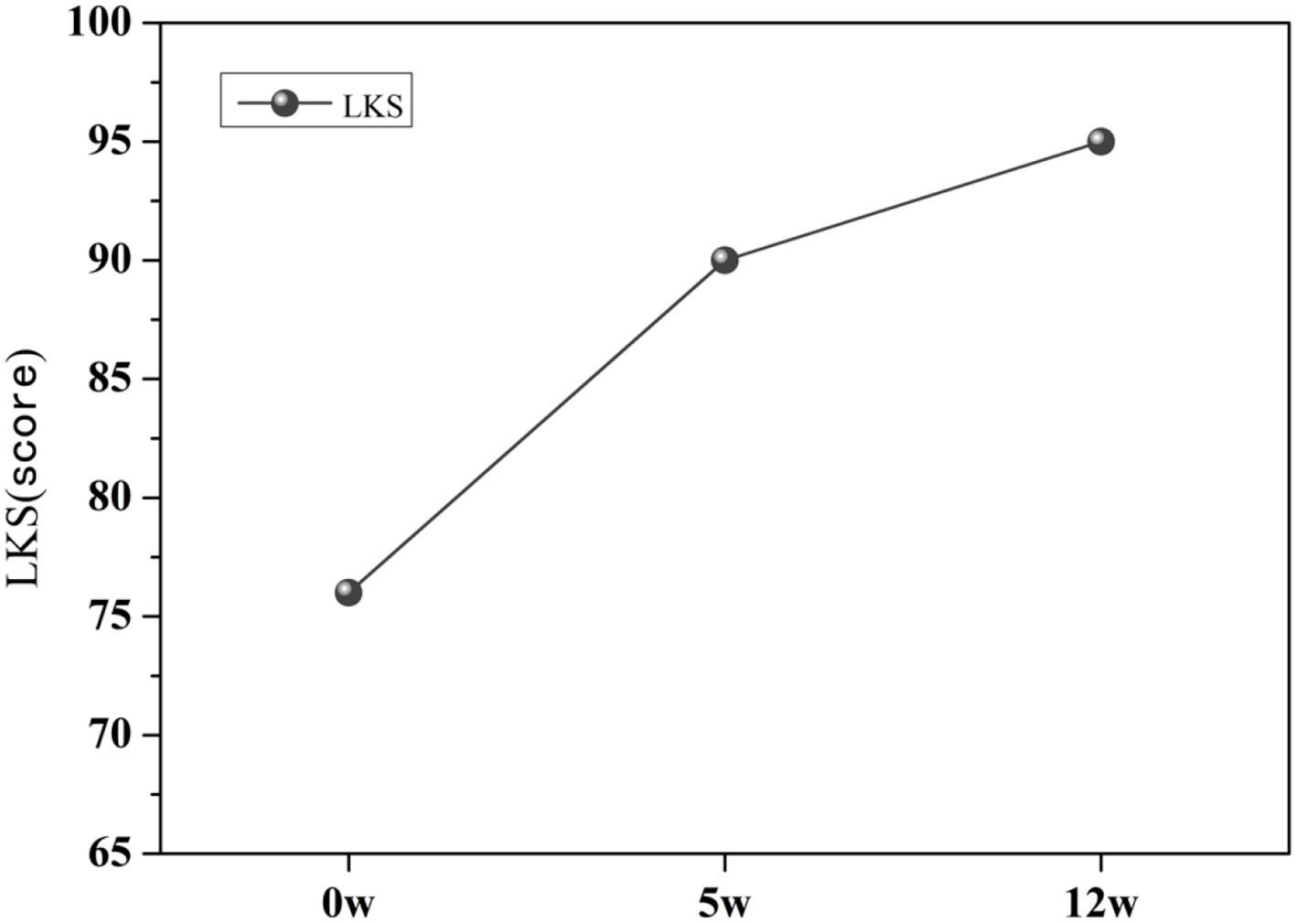
LKS score at pre-intervention, mid-intervention, and post-intervention.

Before exercise intervention, the patient's LKS score was 76 points (Figure 3). After the 5th week of exercise intervention, the patient's LKS score increased to 90 points, and after the 12 week of exercise intervention, the patient's LKS score was 95 points. The knee joint function was significantly improved.

### VAS Results

**Figure 4:**
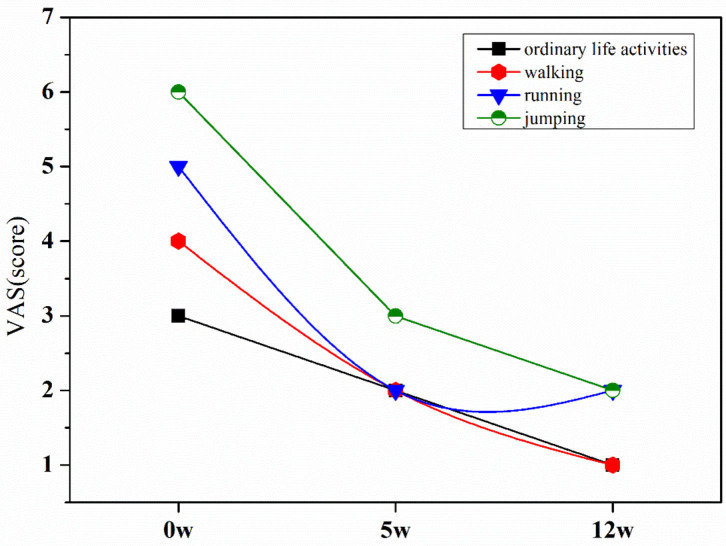
VAS score at pre-intervention, mid-intervention, and post-intervention.

Before the exercise intervention, the patient's ordinary life activities VAS score was 3/10, walking was 4/10, running was 5/10, and jumping was 6/10 (Figure 4). After the 5th week of the exercise intervention, the patients' ordinary life activities, walking, and running VAS scores were reduced to 2/10 points, and the jumping VAS scores were reduced to 3/10 points. In the 12th week of the exercise intervention, the patient's pain was significantly improved, ordinary life activities and walking pain were 1/10 points, running and jumping were 2/10 points, and the patient's pain was significantly reduced after the exercise intervention.

### TSK Results

**Figure 5:**
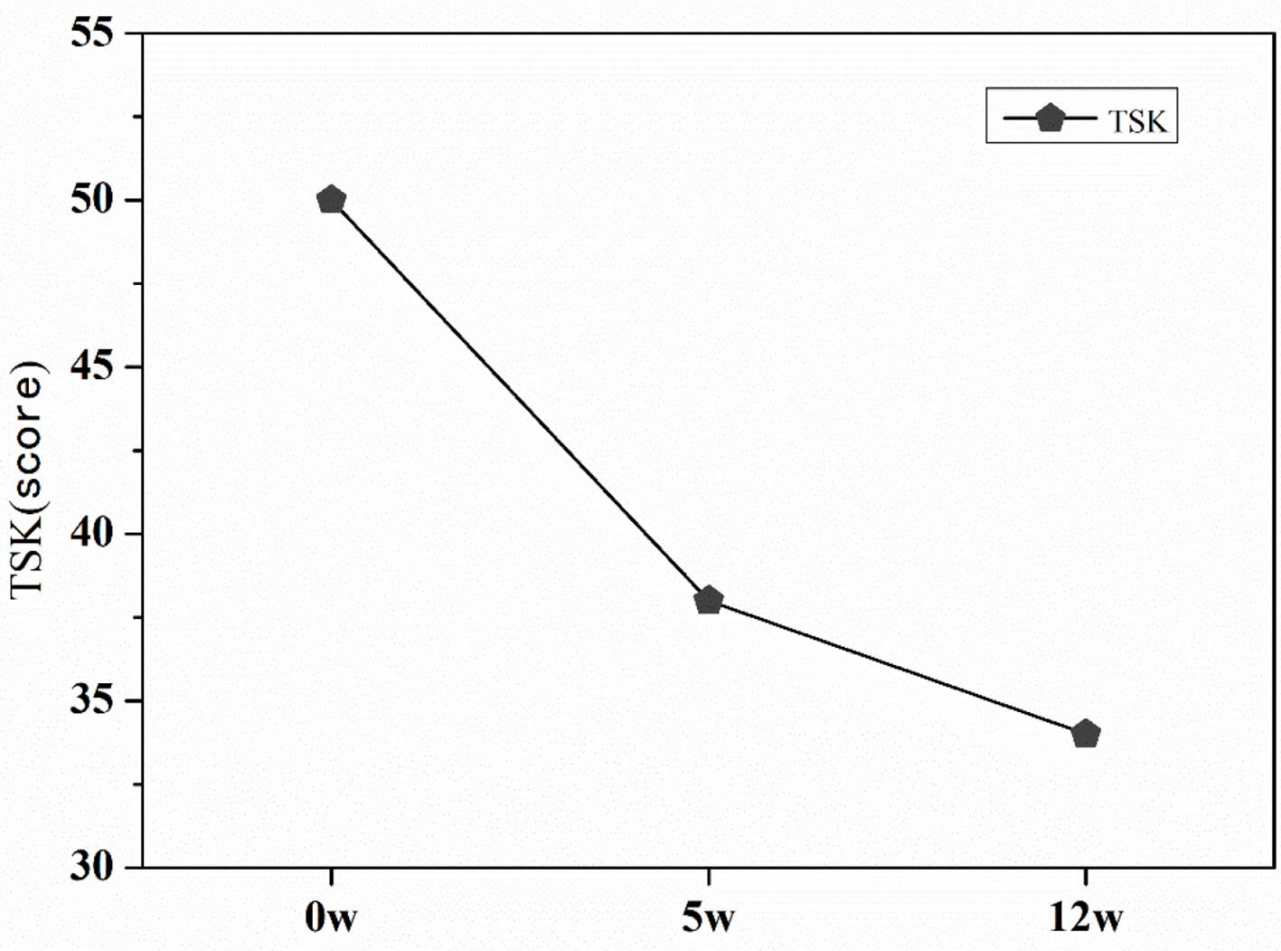
TSK score at pre-intervention, mid intervention, and post-intervention.

Before exercise intervention, the patient's TSK score was 50 points (Figure 5), and the patient had kinesiophobia. After the 5th week of intervention, the patient's TSK score was reduced to 38 points, After the 12 weeks of exercise intervention, the patient's TSK score was 34 points, and the fear of movement was significantly relieved.

## Discussion

This study mainly explored the effects of exercise intervention on the knee joint function of patients with chronic knee pain, the degree of knee joint pain in different states, and fear of movement. The patient's knee joint function, pain, and fear of exercise have been significantly improved by performing exercise intervention for the patient 3 times a week, 60 min each time, for a total of 12 weeks. After 5 weeks of exercise intervention, the patient's knee joint function, pain, and kinesiophobia had been significantly improved. It can be seen that exercise intervention is beneficial to the improvement of the physical function, pain and fear of movement in patients with chronic pain. At the same time, after 12 weeks of exercise intervention, the patient's hydrops of bursae infrapatellaris profunda was significantly reduced. Studies have shown that reduced fluid effusion indicates relief of symptoms. In addition, the patient's active mobility of the hip and ankle joints was improved. Right hip extension-hip abduction-ankle dorsiflexion: 20°-35°-18°, and the left hip extension-hip abduction-ankle dorsiflexion: 19°-34°-15°. At the same time, the patient felt that his physical function was significantly improved, and he was able to face jumping movements in life actively. The study provides a good idea for the patient's current fitness. The normal physiological range hydrops of bursae infrapatellaris profunda is 1–2 mmand the depth of fluid effusion after exercise intervention is 1.5 mm, which belongs to the normal range (Li, 2016). It can be seen that exercise intervention can affect patients so that the symptoms were relieved. At the same time, some studies that were consistent with the results of this study. There is a lot of evidence that exercise intervention can improve the dysfunction and pain of patients with chronic pain. Simultaneously, a review study showed that muscle strength training around the hip joint can effectively improve the lower limb function and biomechanical measures of the knee in patients with chronic knee pain. A systematic review identified strong and high-quality evidence to recommend hip muscle strengthening in the conservative management of patients with knee OA (Raghava et al., 2020). The exercise program of this study also paid special attention to the ideal training of the muscles around the hip joint. At the same time, some studies demostrated that muscle strength of the hip could help restore knee function and relieve pain. Hip and knee strengthening is effective and superior to knee strengthening alone for decreasing pain and improving activity for PFPS (Nascimento et al., 2018). In addition to solving the strength exercises of the muscles around the knee joint of the patient, it also fully combines the functional training in the exercise program. There was research evidence that the gluteus maximus and gluteus medius muscles were more likely to be inhibited in daily life, leading to a decrease in muscle strength, which made the hip joint unstable in the frontal and sagittal planes, resulting in lower walking efficiency, lower limbs and the damage risk increases. At the same time, the gluteus medius muscle plays an important role in maintaining the stability of the lower limb chain in daily support and walking exercises. A study has shown that strengthening hip abductors and external rotators in addition to knees is more efficient, allowing for muscle training while reducing pain and increasing function and muscle strength (Sahin et al., 2016).

Research similar to this study proved that strengthening the hip joint and core strength is more effective than simply training the muscle strength around the knee joint to improve chronic knee pain. The study conducted a randomized controlled trial on 199 patients with patella pain syndrome. The results showed that both the HIP and KNEE rehabilitation protocols produced improvements in PFP, function, and strength over 6 weeks. Although outcomes were similar, the HIP protocol resulted in earlier resolution of pain and greater overall gains in strength compared with the KNEE protocol (Ferber et al., 2015). In addition, gluteal muscle activity under functional tasks (e.g., single-leg squats and other weight-bearing conditions) can affect lower limb mechanics and the arrangement of the kinematic chain (DeJong et al., 2020) When standing on one leg, the activation and contraction of the gluteus medius can prevent the contralateral pelvis from loweringwhich is essential for the stability of the pelvis when walking (Flack et al., 2012). At the same time, weakness or insufficient activation of the gluteus medius during weight-bearing can cause internal rotation of the femur, resulting in lateral displacement of the patella and patellofemoral joint (Semciw et al., 2013; Petersen et al., 2014). The gluteal muscles are vital for the stability of the knee joint. For example, in the exercise program, the strength exercises emphasize the gluteal muscles, involving the side-lying straight leg lift, hip bridge, clam opening and closing movements.

In addition, there are also researches on sports intervention on fear of movement, fear-avoidance, and panic disorder. A systematic review showed that there was very low to low-quality evidence that exercise training was effective for reducing fear-avoidance; exercise training may be more effective than no intervention for reducing fear-avoidance (Hanel et al., 2020). Furthurmore, there is very low-quality evidence that exercise training cannot reduce fear avoidance. Still, the research also pointed out that more high-quality research was needed to verify the influence of exercise intervention on the psychology of exercise fear. Some studies suggested that exercise intervention can alleviate the fear of patients with chronic pain. For example, research evidence showed that 12 weeks of Pilates exercise can effectively treat disability, pain and kinesiophobia in patients with chronic non-specific low back pain(Cruz-Díaz et al., 2018). At the same time, the study found that Major changes in disability and kinesiophobia were observed at 6 weeks of intervention with no significant difference after 12 weeks. Studies have shown that panic disorder can cause pain-related fear, pain catastrophe, fear of activities, anxiety/depression and other bad emotions, which affect rehabilitation, surgical efficacy and quality of life. But there is no systematic and standardized intervention method at the early stage of rehabilitation. It has been found that panic disorder can better prevent and intervene in patients with chronic pain and exercise fear (Deng et al., 2020). The goal should be to reduce pain intensity and strengthen health beliefs to promote patient recovery (Larsson et al., 2016). In this case, the patient was afraid of movement, and was more afraid of jumping. In the exercise program's design, the patient's particular situation is fully considered, and jumping-type movements are trained to re-establish the patient's understanding of jumping-type movements and overcome fear. In the exercise program, bipedal jumping and single-leg jumping movements were specially added to allow patients to rebuild their confidence in jumping movements and reduce the degree of fear of movement. For the recovery of sports injuries to relieve symptoms, it is necessary to pay attention to functional recovery. In particular, old injuries need to solve functional problems, and it is also necessary to pay attention to the patient's psychological disorder (e.g., kinesiophobia). The method of functional sports rehabilitation is obviously the main method to solve motor dysfunction. In the rehabilitation of sports injuries, the body needs to be regarded as a whole, and the reason can be found from the perspective of the power chain to better enhance the scientificity, safety and rationality of training.

## Limitations

A typical case study cannot draw causality. Due to the particularity of each person, the design of the plan depends on the judgment and personal experience of the practitioner, so in the follow-up research, the design of the plan should be tailored to individual circumstances. As a result, the application of this exercise program has certain limitations, only for a patient with a chronic old patella fracture.

## Implications

Our research concluded that exercise intervention can effectively alleviate the function, pain and phobia of patients with chronic knee pain, which is similar to other studies. Future research can be improved on the sports rehabilitation program, consider the training of other joints for a certain part of the injury, and the hip, ankle and core area for the rehabilitation of the knee joint. At the same time, a long-term follow-up of the patient's symptoms. In addition, in-depth study of the mechanism of exercise intervention to improve patient function, pain and kinesiophobia.

## Conclusion

In this case, exercise intervention can effectively improve knee function, pain and kinesiophobia in patients with old patella fractures. Detecting panic disorder in the early stage of rehabilitation can better prevent and intervene in patients with chronic pain and fear of movement.

## Data Availability Statement

The datasets presented in this article are not readily available due to patient privacy reasons. Requests to access the datasets should be directed to Li Peng, 804455169@qq.com.

## Ethics Statement

The studies involving human participants were reviewed and approved by the Ethics Committee of Physical Education of Southwest University School (approval no. SWU-20180304-C1). The patients/participants provided their written informed consent to participate in this study. Written informed consent was obtained from the individual(s) for the publication of any potentially identifiable images or data included in this article.

## Author Contributions

LP was responsible for experimental design and financing. YS and LH were responsible for collecting data and writing the article. HL was responsible for sports intervention and revision of the article. SC was responsible for data collection and processing and drawing. All authors contributed to the article and approved the submitted version.

## Funding

This study was supported by the Chongqing University of Posts and Telecommunications Social Science Fund Project (Grant No. K2020-84) and Central University Fund (Grant No. SWU1909105).

## Conflict of Interest

The authors declare that the research was conducted in the absence of any commercial or financial relationships that could be construed as a potential conflict of interest.

## Publisher's Note

All claims expressed in this article are solely those of the authors and do not necessarily represent those of their affiliated organizations, or those of the publisher, the editors and the reviewers. Any product that may be evaluated in this article, or claim that may be made by its manufacturer, is not guaranteed or endorsed by the publisher.
